# In quest for leukemia initiating cells in AML

**DOI:** 10.18632/oncoscience.394

**Published:** 2018-02-23

**Authors:** Maria De Grandis, Stéphane JC Mancini, Michel Aurrand-Lions

**Affiliations:** Aix-Marseille University, CNRS, INSERM, Institut Paoli-Calmettes, CRCM, Marseille, France

**Keywords:** JAM-C, adhesion, bone marrow niches

Leukemic stem cells (LSC), also referred as leukemia-initiating cells (LIC), represent a rare subpopulation of leukemic cells that possess stem cell properties distinct from bulk leukemic cells. Such properties include leukemia initiation (the ability to engraft and to reconstitute the heterogeneous leukemia disease), self-renewal (the ability to transfer disease into secondary/tertiary recipients) and drug resistance. In Acute Myeloid Leukemia (AML), LSC were identified more than 20 years ago as a population of leukemic cells with the CD34^+^CD38^−^ immunophenotype [1, 2]. However, more recent studies have challenged this result and have shown that LSC are also present in other phenotypically defined compartments [3, 4]. In their study, Ng et al. have demonstrated that expression of a 17 gene signature derived from functionally defined LSC predicts the risk for relapse irrespective of CD34/CD38 expression. This suggests that leukemia initiating activity and leukemic cell chemoresistance is a cellular intrinsic property independent of cell surface phenotype.

In our recent study, we have found that JAM-C expression can be used to enrich the CD34^+^CD38^−^CD123^+^ leukemic compartment in cells initiating and sustaining leukemia [5]. To our knowledge, the level of LSC enrichment (1/63) obtained with combination of CD34, CD38, CD123 and JAM-C has never been reached before. In addition, engraftment and reconstitution of human AML in *NOD.Prkdc^Scid^.Il2rg^Null^* (NSG) mice were highly enriched in JAM-C expressing cells for three patients, suggesting that JAM-C expression was associated to an intrinsic molecular mechanism involved in leukemic stemness maintenance and/or engraftment. Since JAM-C is an adhesion molecule involved in normal HSPC interactions with bone marrow stromal niches through interaction with JAM-B [6], it is not surprising that the adhesive function of JAM-C is also involved in leukemic engraftment. However, isolation of two variant cell lines from KG1 cells raised additional questions with respect to the molecular mechanisms by which JAM-C expression controlled leukemic stemness. Indeed, the KG1 cell line has been established from a patient with AML in 1977 and is maintained in culture without micro-environmental pressure. Although we found only subtle differences in gene expression between KG1 JAM-C^Pos^ and KG1 JAM-C^Neg^ cells, the two variant cell lines differed by their ability to engraft in NSG mice and by the activation status of Src family kinases (SFK). Hyperactivation of SFK was lost upon JAM-C silencing suggesting that JAM-C acts as a signaling molecule in a cell autonomous manner. The correlation between JAM-C expression and SFK hyperactivation was confirmed in cells isolated from blood patient samples. Whether this is due to constitutive signals delivered by JAM-C or autocrine signaling via a secreted ligand remains to be determined. However, our results already indicate that antibodies against JAM-C may be used as a vectorization method to target tyrosine kinase inhibitors to LIC. A second aspect of our work points out to the prognostic value of JAM-C-expressing cell frequency in the blood samples of patients at diagnosis. High frequencies of JAM-C expressing cells are a marker of poor disease outcome independent of the chromosomal and molecular aberrations used to assess patient risk, showing that measuring frequencies of rare leukemic stem cells adds value on previously identified risk groups. Measurement of JAM-C expressing cell frequency could be rapidly generated by flow-cytometry forming the basis of a fast prognostic test for patients. In addition, the systematic increase of JAM-C expressing cell frequency observed at relapse strongly suggests that chemo-resistant leukemic cells (RLC) have the CD34^+^CD38^−^ CD123^+^JAM-C^+^ immunophenotype in patients.

This result is in apparent contradiction with the recent landmark study by Farge et al [7]. In this study, the authors have demonstrated that RLCs do not express a specific immunophenotype, but correspond to preexisting or selected cells able to adapt their energetic metabolism toward a high OXPHOS phenotype in response to Ara-C. Accordingly, modulation of mitochondrial OXPHOS status markedly affects the anti-leukemic effect of Ara-C on patient derived xenografts (PDX) in busulfan conditioned mice. A significant increase in CD34^+^CD38^−^ cells after Ara-C treatment was observed in patients having the lowest level of this immature population. In contrast, PDX from patients having more than 10% of CD34^+^CD38^−^ cells rather showed a decrease in cells presenting such a phenotype after Ara-C treatment, suggesting that RLC cannot be defined based on CD34 and CD38 expression. This is in agreement with our results showing that frequencies of CD34^+^CD38^−^ cells are not significantly altered in paired patient samples at diagnosis and relapse. However, frequencies of JAM-C-expressing LSCs, which represent only a minor fraction of CD34^+^CD38^−^ cells, are systematically increased at relapse [5]. In addition, the few patient samples having less than 1% of CD34^+^CD38^−^ cells that we have analyzed showed the highest frequencies of JAM-C expressing cells. Such cells express a pro-adhesive gene signature that could facilitate leukemic cells interaction with bone marrow niches in which metabolic alterations and clonal evolution would then take place.

Collectively, these results underscore the necessity to dissect LSC (or LIC) heterogeneity with respect to phenotypic, genomic or metabolic diversity in order to better understand the molecular mechanisms driving chemoresistance in AML. Indeed, it is likely that RLC and LSC represent two overlapping entities and that multistep alterations occurring at the genomic or metabolic levels lead to LIC heterogeneity reflected by phenotypic diversity. Current and future studies will likely define the common features between LIC and RLC purified at the single cell level to pave the way toward new therapeutic strategies aiming at RLC eradication.
